# Extrusion of Heterogeneous Filament‐like Structures: A New Paradigm in Fabricating Soft Mechanical Gradient with Long Span

**DOI:** 10.1002/smsc.202500234

**Published:** 2025-05-20

**Authors:** Akanksha Pragya, Tushar K. Ghosh

**Affiliations:** ^1^ Fiber and Polymer Science Program Department of Textile Engineering Chemistry and Science Wilson College of Textiles North Carolina State University Raleigh NC 27606 USA

**Keywords:** co‐extrusion, expandable microspheres, mechanical gradient, strain distribution, soft‐hard interface

## Abstract

Soft‐to‐hard material interfaces found in multimaterial systems, such as microelectronics, prosthetics, body armor, and soft robotics, often suffer from mechanical mismatches that compromise their structural integrity overtime. These mismatches occur due to significant differences in mechanical properties, such as stiffness, between soft materials (e.g., polymers and biological tissues) and hard materials (e.g., metals and ceramics). In this study, an extrusion‐based approach is presented to fabricate continuous stiffness gradient materials using polydimethylsiloxane and thermoplastic expandable microspheres (EM). Morphological characterization shows the intended distribution of EM content along the length of the filament and the corresponding variation in tensile and bending stiffness. The gradient mechanical properties can be tuned by varying the EM expansion temperature. Compared to traditional fabrication techniques, this method allows for precise control over gradient magnitude and span, even post‐fabrication, offering greater flexibility for various applications. This work demonstrates a scalable and efficient solution for mitigating the mechanical mismatch at soft–hard material junctions, offering the potential for advanced material design in both industrial and biomedical applications.

## Graphical abstract

An extrusion‐based technique to engineer a stiffness gradient interphase material that seamlessly bridges soft‐to‐hard interface without strain localization in multimaterial systems is presented. The gradient is obtained by spatial control of the distribution of reinforcing expandable microspheres in polydimethylsiloxane. This strategy offers a solution for mitigating mechanical mismatches in flexible electronics and others.

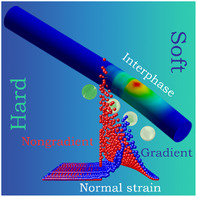

## 1 Introduction

**Figure 1:**
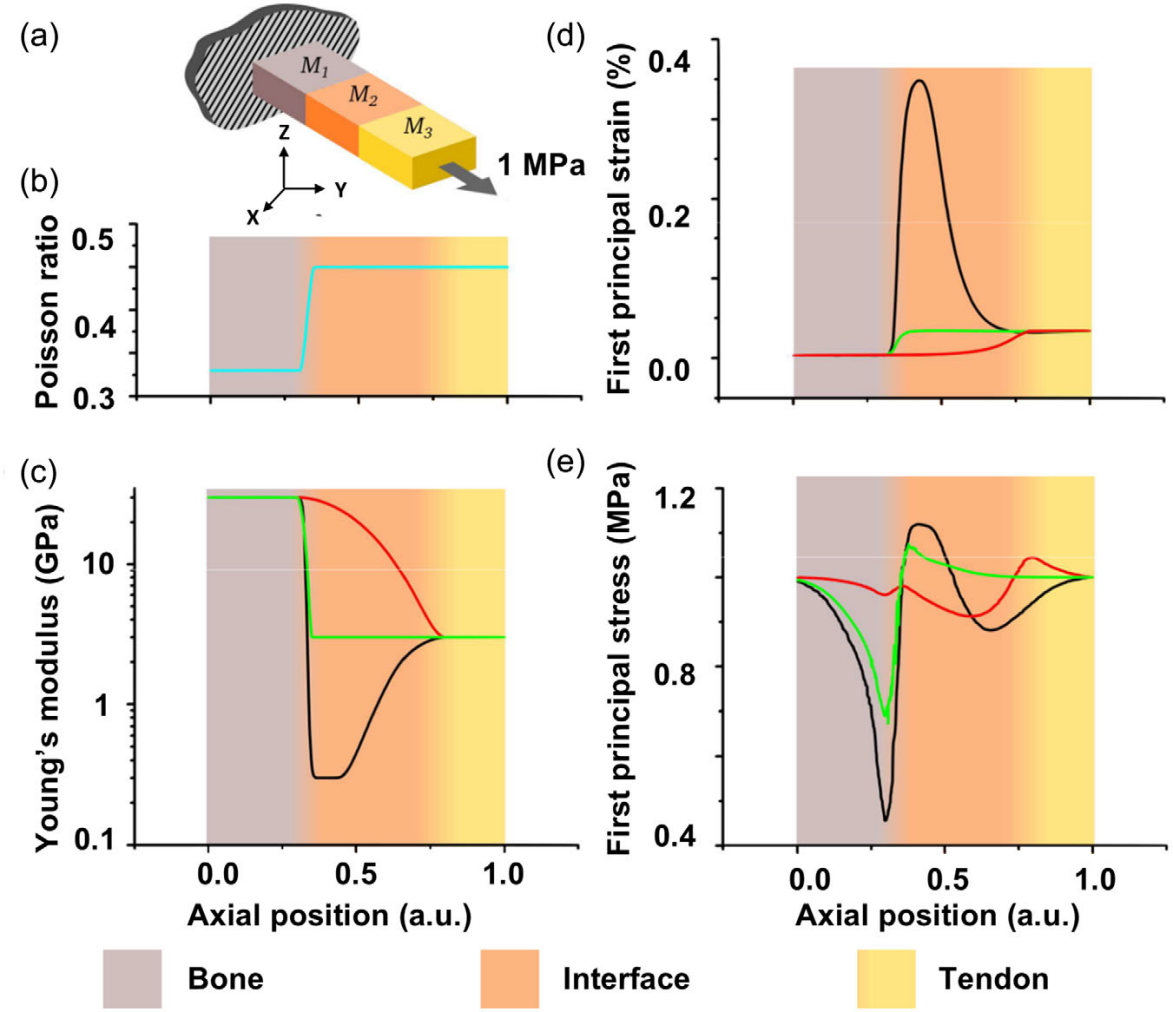
Simulation of mechanical response of a) model bone*–*tendon (M1*–*M3) attachment: a hard*–*soft attachment with an interphase (M2). The model is subjected to an axial tension. Different interfaces are investigated by varying the b) Poisson ratio and c) Young's modulus of M2. Moduli of M1 and M3 are fixed at 30 and 3 GPa, respectively. The material properties of the attachment are shown in red, green, and black for a graded transition, a direct attachment, and a compliant attachment, respectively. In d,e) two calculated parameters related to the stability of the attachments are shown. The axial position in all plots is shown in arbitrary units. Reproduced with permission.^[^ ^19^ ^]^

Nearly every functional device or system surrounding us comprises a combination of hard (e.g., metals, ceramics, etc.) and soft (e.g., rubber, textiles, biological tissues, etc.) materials. Such multimaterial systems form soft‐to‐hard interfaces, i.e., junctions where two mechanically (or otherwise) mismatched phases meet. Soft and hard materials at these interfaces may exhibit mismatched mechanical,^[^ ^1^, ^2^ ^]^ thermal,^[^ ^3^, ^4^ ^]^ or chemical/biochemical properties, among others.^[^ ^5^ ^]^ Issues related to the mechanical mismatch, in particular, have been a long‐standing area of concern, spanning both the academic and commercial space, entailing diverse fields of application, e.g., industrial tools, electronics, aerospace, tissue engineering, automobiles, body armor, etc.^[^ ^6^, ^7^, ^8^, ^9^, ^10^, ^11^, ^12^, ^13^, ^14^ ^]^ Differences in mechanical properties, such as stiffness (or modulus), often lead to strain localization and discontinuity in strain fields at the mismatched interface.^[^ ^12^, ^15^, ^16^, ^17^, ^18^ ^]^ The concomitant stress singularities at the interface can cause it to weaken over time and may hamper the structural integrity of the system. To demonstrate the point, Rosetto examined the mechanical response of a bone–tendon attachment at the interface using a continuum model and reported that the difference in strain levels of the two tissues results in a stress divergence, see **Figure** **1**.^[^ ^16^, ^19^ ^]^

In principle, mechanical mismatches can manifest at macro‐, meso‐, micro‐, or nanoscale, and even at the molecular level.^[^ ^2^ ^]^ Even homogenous systems like a randomly distributed polymer–particle nanocomposite have several micro‐/nanoscale mismatches observed at the soft polymer‐rigid particle interface.^[^ ^20^, ^21^ ^]^ In the present study, we focus on mechanical mismatches occurring at a macroscopic scale, such as those in soft electronics,^[^ ^2^, ^22^ ^]^ bioelectric interfaces,^[^ ^23^ ^]^ limb prosthetics,^[^ ^14^, ^24^ ^]^ dental implants,^[^ ^25^ ^]^ body armor,^[^ ^26^ ^]^ and bonded (similar or dissimilar) joints.^[^ ^27^, ^28^ ^]^ In all these cases, macroscopic mechanical mismatches occur when the soft polymers and biological tissues, etc., form interfaces with hard materials such as metals, ceramics, and structural composites, etc.

**Figure 2:**
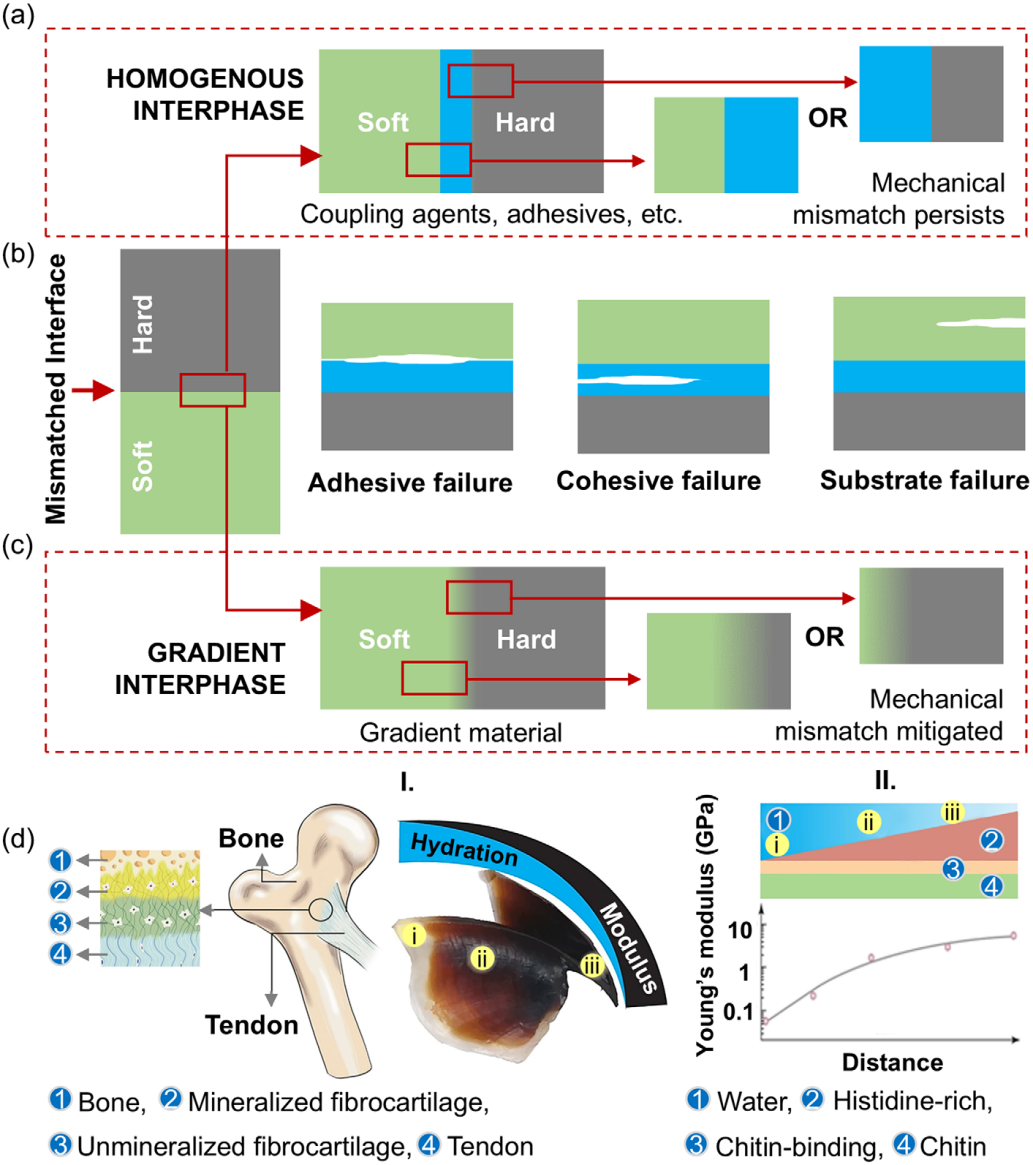
Joining techniques for a soft‐to‐hard interface: a,b) Homogenous interphase—(a) use of homogenous interphase material forms new interface(s) that still includes mismatch; (b) examples of various failure modes of metal–nonmetal joined via polymer adhesives are shown, and c,d) Gradient interphase—(c) the use of gradient interphase materials can either minimize or eliminate mismatches at the new interface; and (d) Examples of gradient interphases in nature, such as (I) the tendon‐to‐bone junction^[^ ^115^ ^]^ and (II) the squid's beak,^[^ ^116^ ^]^ are shown. (I) Adapted with permission^[^ ^115^ ^]^ under terms of the Creative Commons Attribution 4.0 International. Copyright 2021 The Authors. Published by Elsevier Ltd. (II) Adapted with permission.^[^ ^116^ ^]^ Copyright 2015, Springer Nature America, Inc.

The presence of interface and interphase between the filler and the matrix is well‐defined in particle polymer composites.^[^ ^29^ ^]^ In the case of other multimaterial systems, the same definitions can be adopted. While inter*face* is the separation surface of zero thickness between different materials, inter*phase* represents the volume or region between two phases, where a gradual transition of material properties occurs from one material to another into the bulk.^[^ ^30^ ^]^ Commercial adhesives,^[^ ^31^, ^32^ ^]^ coupling agents,^[^ ^33^, ^34^ ^]^ and chemical binders^[^ ^4^ ^]^ have been used as inter*phase* materials to mitigate the issues of mechanical mismatches at various scales. Of these, adhesives are applicable at the macroscopic scale, while the other two are suitable as microscopic interphase material. Even with the best‐performing adhesives, the new interfaces (formed on either side of the inter*phase*) may still have a mechanical mismatch, **Figure** **2**a. Abrupt change at these interfaces can cause failure due to debonding at the interface (adhesive failure), failure of the interfacial adhesive (cohesive failure), or failure of the soft phase (substrate failure),^[^ ^35^, ^36^, ^37^, ^38^, ^39^, ^40^ ^]^ as illustrated in Figure 2b. This is an inherent issue with homogenous inter*phase* materials. However, nature provides an excellent design solution to this issue. Biological species contain a plethora of instances where soft and hard materials, with up to three to four orders of magnitude of mechanical mismatch, are smoothly connected together to support complex functions.^[^ ^41^, ^42^ ^]^ This is enabled by gradual changes in the mechanical properties across the inter*phase*.^[^ ^41^, ^42^ ^]^ Gradient design between soft tissues and mineralized phases enhances mechanical properties, such as load‐bearing, toughness, and resistance to damage. These gradients occur through variations in material composition and structure.^[^ ^41^, ^42^ ^]^ For example, the squid beak features a modulus gradient that facilitates force transfer from the stiff beak to the soft mouth tissues, while the mussel byssus exhibits a hardness gradient aiding bioadhesion. Similar gradients are found in human bone–tendon junctions, providing fracture resistance through a gradual increase in mineralization. The mechanical gradient at the tendon–bone interface in the shoulder joint helps mediate effective load transfer from the soft tendon (Young's modulus ≈0.05–0.5 GPa) to the hard bone (Young's modulus ≈15–175 GPa) without damaging the joint.^[^ ^42^, ^43^, ^44^, ^45^ ^]^ These gradients are also present in other biological structures, such as spider fangs, which display stiffness variations due to ion concentration gradients, and human teeth, where the dentin–enamel junction exhibits a transition in mineral concentration and collagen orientation, ensuring toughness and crack resistance during chewing.^[^ ^41^, ^42^ ^]^ These natural gradient designs optimize mechanical performance by gradually transitioning from dense, stiff mineralized structures to soft, flexible tissues.^[^ ^41^, ^42^ ^]^ They offer excellent examples to learn from and create design solutions for man‐made systems with soft–hard interfaces. Contrary to a homogenous inter*phase* material, a mechanically gradient inter*phase* suppresses or mitigates mismatches at the new interface(s). The absence of abrupt changes in local properties allows them to bridge the soft and hard phases seamlessly, see Figure 2c,d.

**Figure 3:**
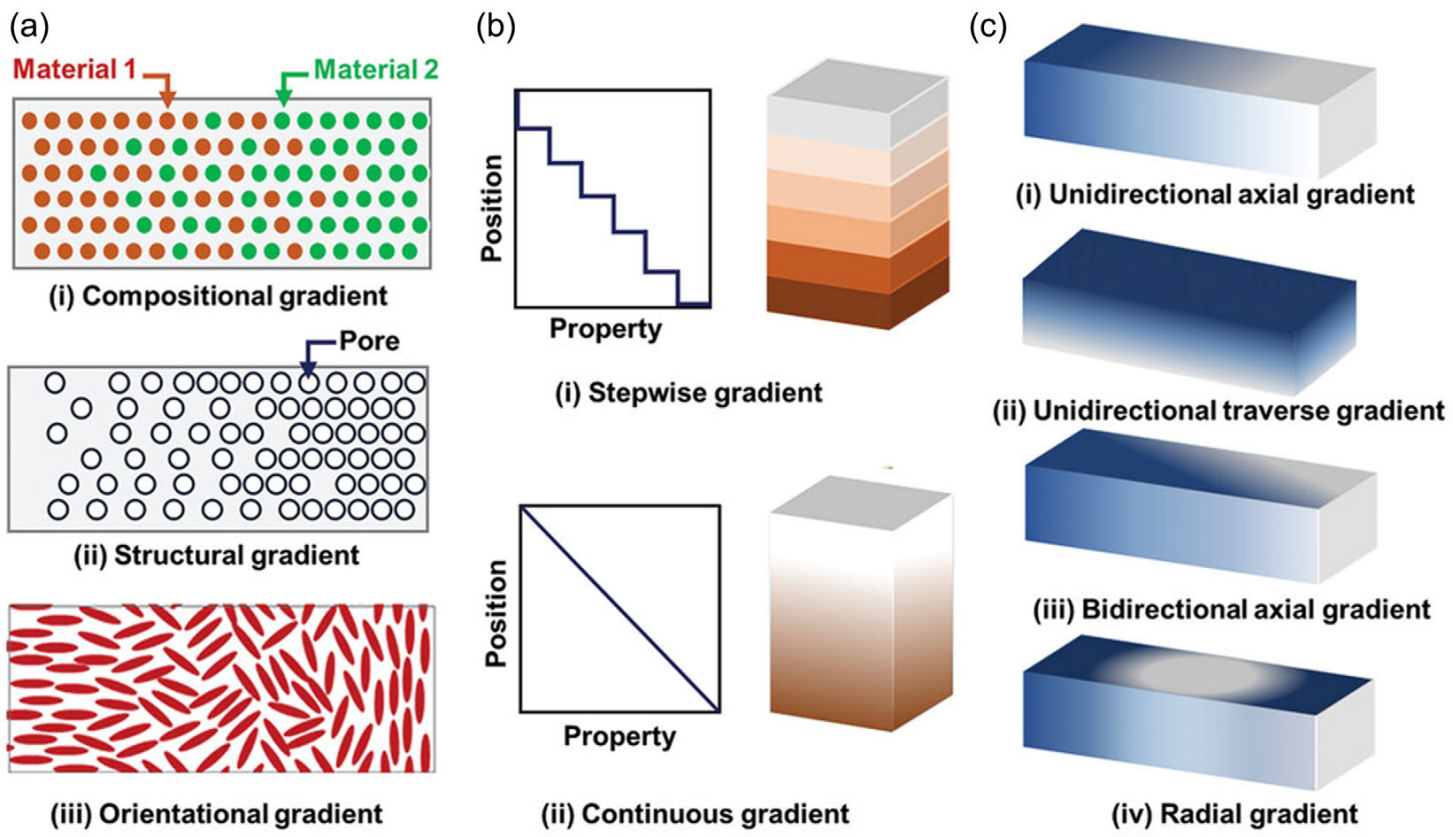
Schematic illustrations of a) different routes for the preparation of functionally gradient systems: (i) compositional, (ii) structural (e.g., pores), and (iii) orientational (e.g., high aspect ratio particles); b) The gradient property transitions: (i) stepwise and (ii) continuous; and c) Directions of gradient: (i) unidirectional axial, (ii) unidirectional transverse, (iii) bidirectional axial, and (iv) radial. Reproduced with permission.^[^ ^41^ ^]^ Copyright 2023 The Authors. Advanced Materials published by Wiley‐VCH GmbH.

Mechanically gradient materials (or structures) can be fabricated by changing one or more of the composition, morphology, and geometry of the material to engender gradual changes in mechanical properties, often stiffness, along a given dimension, see **Figure** **3**. For years, these approaches have been used to develop gradients for specific applications.^[^ ^41^, ^46^, ^47^ ^]^ Several fabrication routes based on the systematic manipulation of composition, structure, or geometry to engender gradient distribution of monomer/cross‐linker, particle size or orientation, porosity, degree of crystallization, and manipulation of cross‐sectional geometry, thickness, etc., have been explored.^[^ ^41^, ^42^ ^]^ However, creating stiffness gradients from soft materials are often accompanied by the difficulty in controlling material distribution during fabrication (e.g., interfacial mixing^[^ ^48^, ^49^ ^]^ magnetic‐force rearrangement,^[^ ^50^ ^]^ etc.). Given these challenges, most publications have reported limited gradient magnitude (degree of change of gradient property) or span (distance over which gradient occurs),^[^ ^51^, ^52^, ^53^, ^54^ ^]^ which in turn limits the scope of application in such gradients. For example, the application of several short‐length soft gradients is mostly limited to tissue engineering.^[^ ^51^, ^55^, ^56^, ^57^ ^]^ While a small gradient span might be suitable for a limited number of specific end uses, it is necessary to develop simple fabrication strategies that can support larger and smaller gradients alike to cater to a wide range of applications.

Materials and technologies to manage interfacial problems are becoming more important with the increasing integration of soft and hard materials in systems such as wearables and soft robotics designed for the human environment. However, merely having a gradient interphase is not enough. These interphases need to be robust, flexible, and stretchable and should have reasonable strength to bear repeated deformation (bending, flexing, stretching, etc.) while being used. In this article, a method of manufacturing a filament with controllable heterogeneous materials distribution and a consequent mechanical gradient structure is reported. Specifically, we disclose a single‐step in‐line mixing and extrusion of a soft, long, and robust filament with a stiffness gradient based on commercial elastomer polydimethylsiloxane (PDMS) and thermoplastic expandable microsphere (EM). While the PDMS is an easily processable, commercially available, and thermally and chemically stable option, the EMs are commercially available blowing agents with core–shell structures, the core is made of a volatile hydrocarbon and the shell is made of thermoplastic. Upon heating above the glass transition temperature of the thermoplastic shell of the EM, the particle undergoes drastic volume expansion as the volatile hydrocarbon pushes against the softened shell.^[^ ^58^, ^59^ ^]^ By incorporating appropriate EM fillers in a PDMS matrix, a composite with the desired mechanical properties can be obtained.^[^ ^59^, ^60^, ^61^, ^62^ ^]^ The in situ expansion of these EM within the cross‐linked PDMS network causes further immobilization of the polymer chains and increases the stiffness of the composite based on the EM content and its extent of expansion within the composite.^[^ ^59^, ^60^, ^61^, ^62^ ^]^ Based on this premise, we have attempted to create a stiffness gradient structure by spatially distributing EM in a given volume. Since the size of the EM can be altered by adjusting temperature during thermal expansion,^[^ ^59^, ^62^ ^]^ its interaction with the matrix polymer can be further controlled to fine‐tune the magnitude of stiffness gradient even after the fabrication is complete and the matrix is fully cross‐linked. Postfabrication tunability of gradients is an enabling technology for the batch production of gradients for specific end‐use requirements.

In this study, we have employed extrusion as a highly flexible route to controllably fabricate structures with the desired span and magnitude of gradient. Extrusion is a commonly used method in shaping (fibers, films, etc.) polymer melts or solutions (often with additives) in high‐volume manufacturing.^[^ ^63^, ^64^, ^65^, ^66^, ^67^, ^68^, ^69^ ^]^ Extrusion‐based gradient formation is desirable as it can support rapid fabrication of high‐resolution (continuous) gradients with precision.^[^ ^55^, ^56^ ^]^ We shape the stiffness gradient EM‐elastomer composite into a high‐aspect ratio filamentous form akin to a textile yarn, particularly suited for applications that require a continuous and gradual stiffness transition along the length of a wire‐like structure. Once rendered electrically conductive, such structures have potential utility in various applications, including electronic textiles in connecting soft textile‐based electrical devices (sensors, etc.) to hard electronic components (power supply, etc.),^[^ ^70^, ^71^ ^]^ in medical devices such as catheters and guidewires having a flexible distal tip region that transitions to a stiffer main shaft,^[^ ^72^ ^]^ in tissue engineering as soft scaffolds wherein axial stiffness gradient enables multiple cell types differentiation and the subsequent tissue development,^[^ ^73^, ^74^ ^]^ and many more.^[^ ^75^, ^76^, ^77^, ^78^, ^79^, ^80^, ^81^ ^]^ We investigate the gradual variation of mechanical and morphological properties, both local and global, to validate the gradient properties in the filament. We believe the proposed fabrication strategy provides new insights and possibilities for designing multimaterial structures using EM particles as a novel, highly tunable, and scalable approach to creating stiffness gradient filaments and others as bridging materials to accommodate soft and hard properties in many emerging applications, such as soft electronics and robotics, autonomous actuators, and tissue engineering.

## 2 Results and Discussion

**Figure 4:**
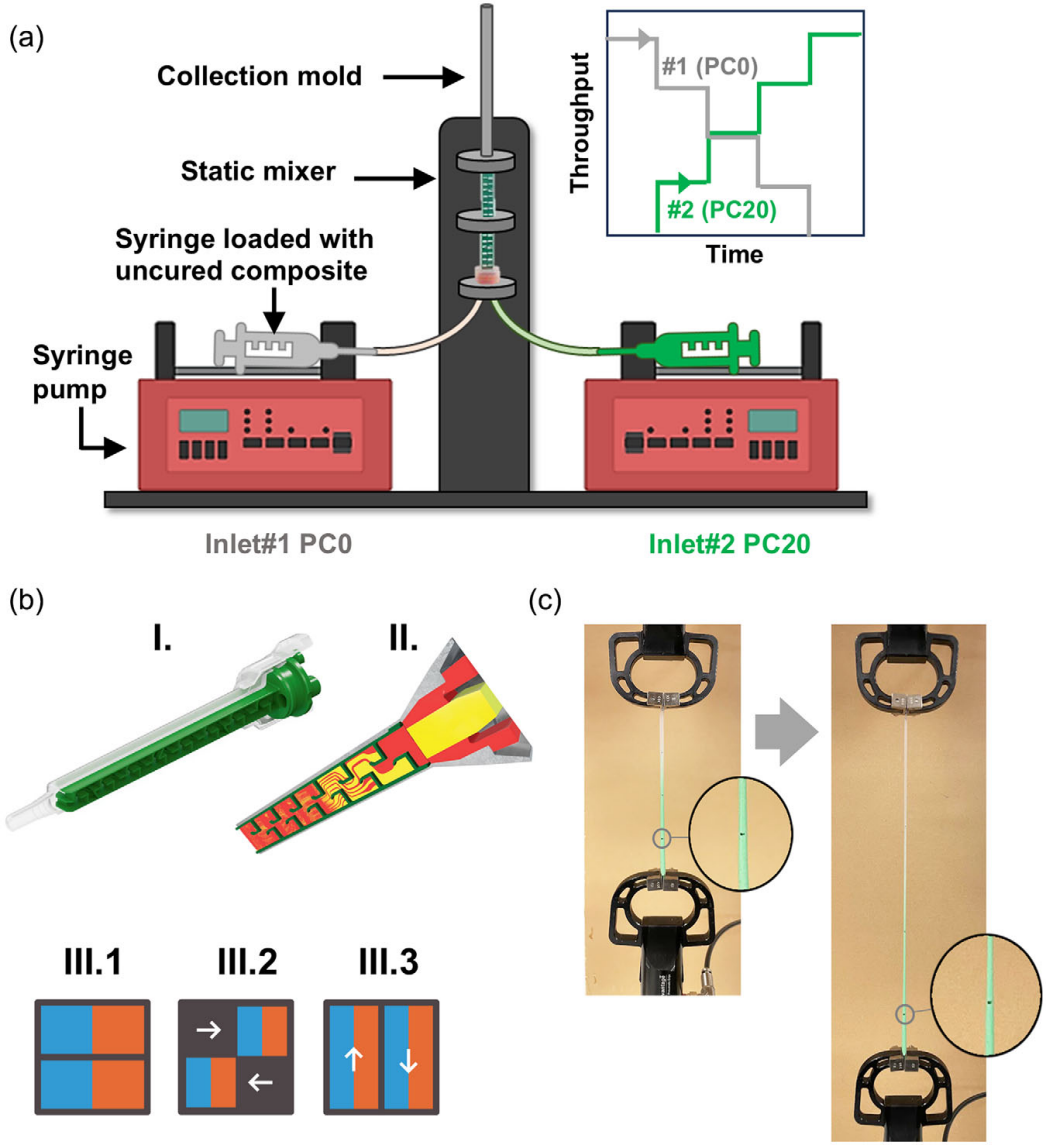
a) Schematic of the co‐extrusion setup, b) flow in a Quadro‐mixer: I. Longitudinal‐section of the mixer,^[^ ^82^ ^]^ II. Flow of two fluids inside the mixer,^[^ ^117^ ^]^ and III. Mixing sequence of the fluid (uncured EM‐PDMS composite) as it progresses along the mixer: 1. Parting, 2. Squeezing and rearrangement, and 3. Elongation,^[^ ^82^ ^]^ and c) methodology used to measure section‐wise strain distribution in the gradient filament. The insets show the marker (point) used to measure the change in the length of the given section. (b) II and III adapted with permission.^[^ ^82^ ^]^ Copyright 2023, Springer Fachmedien Wiesbaden GmbH, part of Springer Nature.

Gradient filaments were fabricated via a programable extrusion setup by coextruding two PDMS‐based composites with 0 and 20 wt% of EM content (referred to as PC0 and PC20, respectively), as outlined in Section 4. The co‐extrusion system consisted of two syringe pumps connected to a static mixer, which enabled precise control over the relative flow rates and composition of the two uncured composites PC0 and PC20, as shown in **Figure** **4**a. The extrudate was collected in a tubular mold and subsequently cured in a convective oven before being subjected to thermal expansion at 130 °C for 30 min. The resulting filaments were characterized by their morphological and mechanical properties. See Section 4 for more details.

### 2.1 Choice of Static Mixer

Fabrication of a gradient filament involves precise, proportional mixing, and co‐extrusion of the feed materials, i.e., composites of PDMS and EM. Static mixers have been considered one of the most convenient choices for mixing two different materials. Compared to other types, static mixers are claimed to mix two component materials consistently regardless of the operating conditions.^[^ ^82^, ^83^, ^84^, ^85^, ^86^, ^87^ ^]^ Their fixed internal geometry and lack of moving parts facilitate thorough and consistent mixing by creating turbulence as materials flow through the mixer.^[^ ^82^, ^83^, ^84^, ^85^, ^86^, ^87^ ^]^ Among the different types of commercial static mixers (like helical, Quadro, T‐mixer, and X‐grid mixers), the Quadro mixer is commonly used for easily mixable materials that have similar viscosities and fall within a low to medium viscosity range.^[^ ^82^ ^]^ Here, we used a Quadro mixer fitted with 16 mixing elements. The two feed materials from inlets 1 and 2 homogenize within the mixer as they flow through it via a combination of dispersive and convective mixing processes, see Figure 4b.^[^ ^82^ ^]^ While dispersive mixing involves repeated parting of the fluid at the walls of the mixer geometry, convective mixing describes the elongation and folding of the fluid by convective shear forces.^[^ ^82^ ^]^ The geometry of the Quadro mixer combines both processes to generate thin layers of each feed as the mixing progresses. The mixer squeezes, rearranges, and elongates the feed multiple times before it comes out of the outlet.^[^ ^82^, ^83^ ^]^

The degree of mixing in the static mixer depends on several parameters, such as absolute and relative viscosity of feed materials, extrusion speed, mixing ratios, and shear forces etc.^[^ ^82^, ^83^, ^84^, ^85^, ^86^, ^87^ ^]^ To elucidate the effects of these factors and to ensure that the gradient obtained from mixing meets the desired morphological variation (based on the changing of the feed throughputs), the static mixer was evaluated for its mixing efficacy. To this end, the ratio of the throughput rates of the two inlets (inlet 1: PC0 and inlet 2: PC10) of the mixer was varied to obtain composites of net EM contents of 2.5, 5.0, 7.5, and 8.3 wt%, as shown in **Table** **1**. Filaments of homogeneous composites of the same four compositions were prepared by directly mixing the required amount of EM with PDMS Part A and B in a planetary mixer, as mentioned in another study.

**Table 1:** Variation in the mixing ratios of the two inlets and corresponding EM content in the extruded composite.

Throughput ratio inlet 1: inlet 2	Inlet 1 (PC0) throughput [cc min^−1^]	Inlet 2 (PC10) throughput [cc min^−1^]	Extrusion time [min]	Net EM content [wt%]
3:1	0.75	0.25	1	2.5
1:1	0.75	0.75	1	5.0
1:3	0.25	0.75	1	7.5
1:5	0.17	0.83	1	8.3

**Figure 5:**
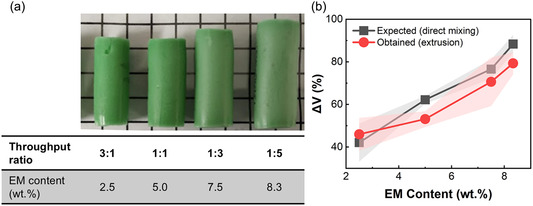
a) Expanded volume of EM‐PDMS composites prepared with different inlet 1:intel 2 throughput ratios and b) comparison between expected volume expansion of the composites obtained via extrusion from the static mixer, and those obtained by direct mixing of the EM and PDMS in a planetary mixer.

The postcuring thermal (volume) expansion of a given expandable microspheres‐polydimethylsiloxane (EM‐PDMS) composite is a direct reflection of its EM content. **Figure** **5**a shows that as the mixing ratios changed due to the relative increase in PC10 (inlet 2), the volume of the expanded composite increased. Furthermore, a comparison of the volume change in extruded and directly mixed composites was found to be very close, see Figure 5b. This established the reliability of the static mixer for the fabrication of EM‐PDMS composite structures used in this study.

### 2.2 Characterization of Gradient Filaments

#### 2.2.1 Morphology

**Figure 6:**
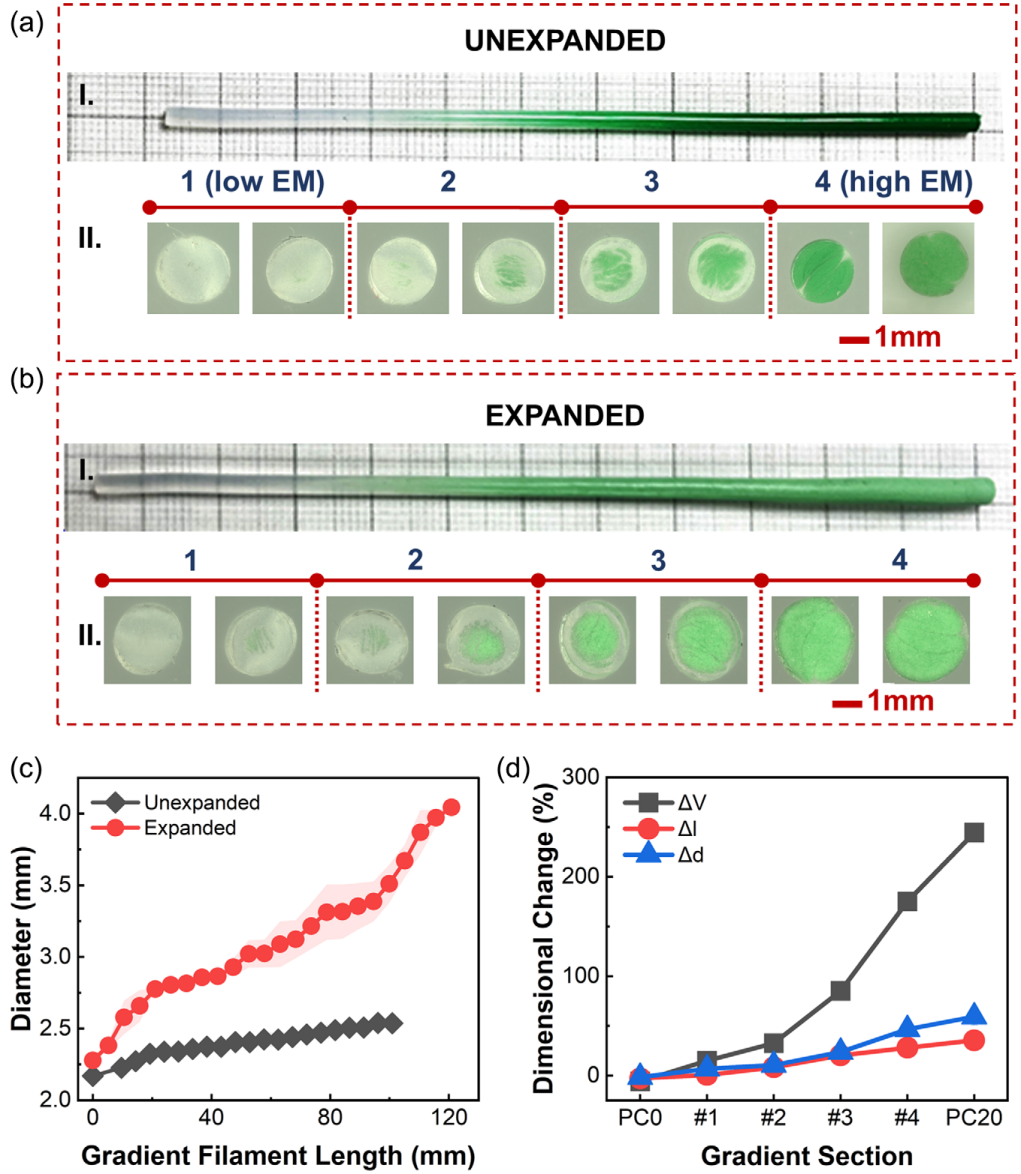
a,b) Gradient filaments in (a) unexpanded and (b) expanded forms: (I) photograph and (II) microscopic images of cross‐sectional variation (at 50× magnification), c) diameter variation along the length, and d) dimensional changes (Δ*V* = volumetric, Δ*l* = longitudinal, and Δ*d *= diametric) in the four sections of the gradient filament. The same for the two feed materials PC0 and PC20 are plotted for comparison.

Mapping of filament morphology should provide one of the strongest pieces of evidence of the gradient nature of the filament. As stated earlier, the high‐aspect ratio filament‐like form factor of the extruded stiffness‐gradient composite is inherently well‐suited to create an inconspicuous interconnect between hard and soft components in electronic textiles or similar applications. Once the efficacy of the static mixer was established, the gradient filaments were extruded, as outlined in Figure 4a and Section 4. As the throughputs of inlet1 and inlet2 changed continuously during co‐extrusion, the composition of the gradient extrudate went from that of PC0 (translucent) to PC20 (stained green). The resultant gradient profile became sharper after thermal expansion due to the dimensional changes caused by the in situ EM expansion (**Figure** **6**a–c). Cross‐sectional images obtained along the filament length show a gradual increase in the EM content, both in the unexpanded and expanded state, see Figure 6a,b. The filament diameter increases as the EM content increases along the length, and this increase becomes more apparent in expanded filaments, as shown in Figure 6c. This is further reflected in the mechanical properties of the gradient filaments, which will be discussed further in Section 2.2.2. Because the size of the EM can be changed by adjusting the temperature during thermal expansion, its interaction with the matrix polymer can be controlled to fine‐tune the stiffness gradient even after the extrusion and full curing of the filament, an ability that hasn't been reported in stiffness gradients until now.

**Figure 7:**
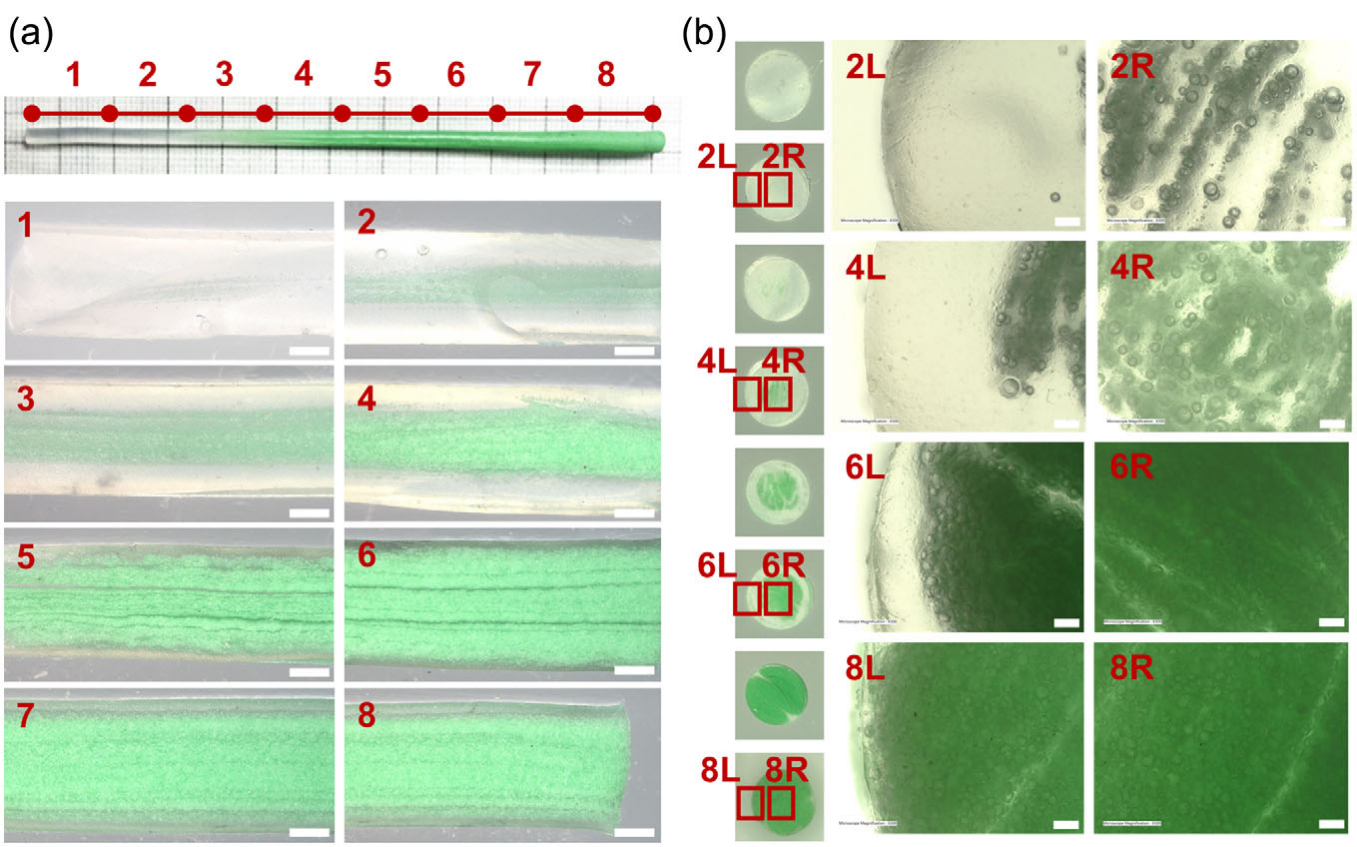
Section‐wise variation in EM distribution in expanded gradient filament: a) longitudinal‐section (magnification 20×; scale bar 1 mm) and b) cross‐section (magnification 50×; scale bar 100 μm) view. The EM content increases from section #1 through #8.

The average dimensional change, due to thermal expansion, in the four equal‐length sections of the gradient filament shows a gradual increase. Figure 6d shows that going from section#1 (low EM content) to section#4 (high EM content), the change in the volume (Δ*V*), length (Δ*l*), and diameter (Δ*d*) gradually increases with the gradual increase in EM content (by increase in PC20 feed material). Evidently, these dimensional changes lie well within the bounds of the two extreme feed material compositions, i.e., PC0 and PC20. The EM‐rich, section #4, experiences significantly less dimensional change compared to PC20. This is likely due to the presence of the unexpandable sheath of PC0 surrounding the EM‐rich core (for more details, see **Figure** **7**). The core‐sheath morphology seen in our filaments is probably a result of the near‐wall effect observed in the laminar flow of fluids within a tube. The flow of high‐viscosity PDMS at a low flow velocity within the static mixers is expected to have resulted in laminar flow. In the absence of turbulence, during laminar flow, mixing occurs through convective (elongation and folding caused by shear forces) and dispersive processes (the repeated separation of the fluid at the walls of the mixing elements) only. Both methods of mixing are ineffective near the tube wall. The fluid velocity not only significantly decreases as it approaches the tube wall (reaching zero velocity directly at the wall surface), but the viscous material (PDMS + EM) in that area is also replaced by less viscous materials (PDMS) due to thermal homogenization and heat transfer between the fluids.^[^ ^88^, ^89^, ^90^ ^]^ This phenomenon is well‐known in co‐extrusion experiments^[^ ^91^, ^92^ ^]^ and in bicomponent extrusion of polymers of different viscosities; the low‐viscosity polymer tends to migrate toward the die‐wall or wrap‐around the higher viscosity polymer.^[^ ^93^, ^94^, ^95^ ^]^ The presence of a core‐sheath morphology in the extruded gradient filaments introduces a fortuitous structural and functional advantage. The thin PDMS sheath surrounding the EM‐laden core acts as a protective barrier, enhancing the filament's mechanical robustness under repeated deformation. This structural feature may be beneficial for applications requiring long‐term mechanical durability. Additionally, if the gradient structure was to be tailored for electrical or thermal conductivity, the PDMS sheath could serve as an insulating layer, ensuring user safety while maintaining functional performance. Alternatively, the sheath may be functionalized to provide electromagnetic shielding. These potential advantages position the core–shell morphology as a useful feature rather than a limitation, offering a new opportunities for application‐driven design of gradient materials.

The variation of cross‐sectional morphology of the gradient filament before and after expansion is interesting. Close‐up views of the longitudinal and cross‐sections of the gradient filament (Figure 7) show visible streaks/layers of the feed materials, which may have developed due to the repeated dispersive and convective mixing. In theory, a Quadro mixer is designed to split each of the two coaxial flows of different materials into two and halve the layer thickness with each mixing element, which in this case would amount to 2^16^ = 65 ,536 material layers. Usually, these layers should no longer be visible to the naked eye by the time they come out of the mixer.^[^ ^82^ ^]^ However, as mentioned above, due to the very small quantity of the extruded volume (≈0.5 cc over ≈100 mm) and the rapidly changing feed throughput within this small volume may have been insufficient for complete homogenization.

**Figure 8:**
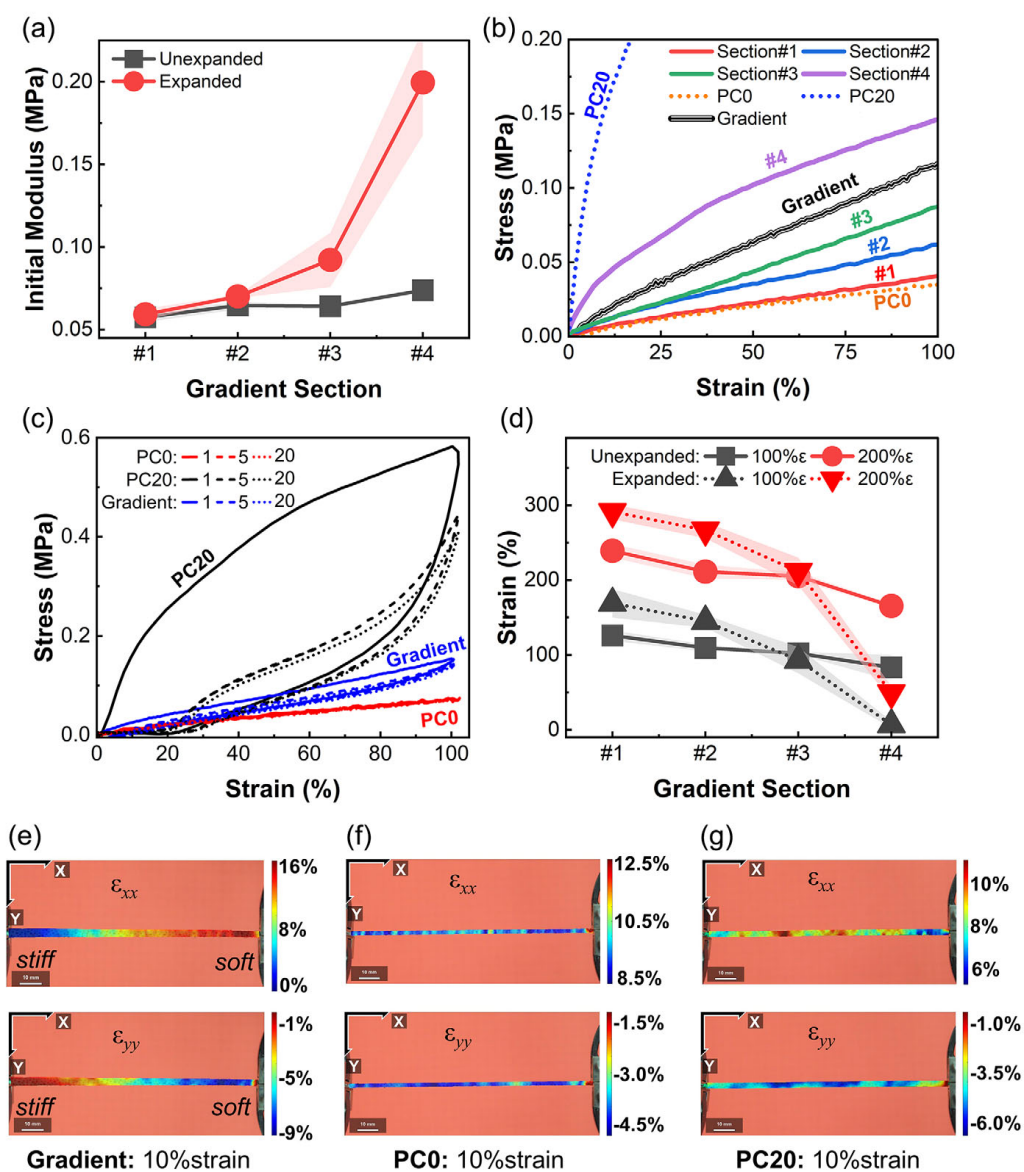
Mechanical property variation in gradient filament section from low (0 wt%) to high (20 wt%) EM content: a) initial modulus of unexpanded and expanded gradient sections, b) tensile stress‐strain curve of expanded gradient sections, gradient filament, and homogenous filaments PC0 and PC20, c) cyclic stress‐strain behavior of gradient filament and homogenous filaments PC0 and PC20 over 20 tensile cycles of 100% strain amplitude (the numbers 1, 5, and 20 in the legend indicate strain cycle number), d) strain distribution in the expanded gradient sections, and e–g) DIC analysis—local strain distribution in the (d) gradient, (e) PC0, and (f) PC20 filaments in the longitudinal (ε_*xx*_) and transverse (ε_*yy*_) direction.

Despite the microstructural inhomogeneities, no visible structural deficiencies were observed in the gradient filaments during mechanical testing. The gradient's mechanical performance remained within the bounds of its feed materials and aligned with the average of its four individual sections (more information in Section 2.2.2 and **Figure** **8**). While minor local variations exist, the overall material distribution tends toward cross‐sectional homogeneity. Thus, the incomplete homogenization does not significantly impact the structural integrity of the gradient filaments for practical applications. The authors realize that this aspect of the extrusion process is an area for further improvement. However, since the focus of this research is on gradient fabrication, any effort toward improving the degree of homogenization was left for further exploration.

#### 2.2.2 Mechanical Properties

The stiffness gradient in this study is fabricated by gradually increasing the EM content along the length during the filament co‐extrusion. The gradient mechanical properties of the filaments were evaluated experimentally using two methods discussed in Section 4. While in the direct method, both unexpanded and expanded filaments were divided into four equal‐length segments for tensile characterization, in the indirect method, the entire expanded filament was evaluated under uniaxial tension and characterized for strain distribution and stress softening along the length.

Embedding an elastomer with nano or microparticles changes its mechanical properties.^[^ ^21^, ^59^, ^96^, ^97^ ^]^ In our previous study, we reported that increasing EM loading increases the stiffness of the composite due to a combination of particle–particle and particle–polymer interactions.^[^ ^59^ ^]^ The filament prepared in this study also showed a gradual increase in the initial modulus from section #1 to #4 with a corresponding increase in EM content (Figure 8a). In the unexpanded state, the initial modulus increases from ≈0.06 to ≈0.08 MPa, creating a stiffness gradient along the filament length. Upon expansion, the magnitude of the stiffness gradient increases significantly from ≈0.06 to 0.2 MPa, more than threefold over a span of ≈120 mm. The data show that a gradual increase in EM distribution along the length results in a corresponding increase in stiffness, thereby creating gradient stiffness in the filament. This also demonstrates the efficacy of the current material choices and fabrication process in tuning the magnitude of stiffness gradient during the post‐fabrication process, such as thermal expansion. Expansion temperature dependance of stiffness has also been reported in other studies.^[^ ^59^, ^62^ ^]^ The tensile stress–strain behavior of the four expanded filament segments is shown in Figure 8b, together with the same for filaments extruded (and expanded) from neat PC0 and PC20, the two extreme compositions of the composite that make up the gradient. As expected, the stress–strain plots of the gradient filament segments are enveloped by the same for PC0 and PC20. Notably, the stress–strain behavior of section #4 (the EM‐rich segment) differs significantly from the neat and homogenous PC20 filament. Once again, the likely reasons are the core‐sheath structure of the gradient filament noted earlier, as well as variation in mixing. The softer PC0 sheath around the PC20 core (as shown in Figure 7) is likely to dampen the mechanical properties of section #4 and restrict its volume expansion (Figure 6d). The stress softening and hysteresis properties of the gradient and homogeneous filaments of two feed material compositions (PC0 and PC20) were also characterized by measuring their stress–strain behavior over 20 tensile cycles of 100% strain amplitudes (Figure 8c). As expected, PC0 (neat PDMS) showed almost no softening beyond the 2nd cycle, while PC20 showed significant softening during the first few cycles. Additionally, while PC0 produced very little nonrecoverable deformation, PC20 displays a large permanent deformation. These observations are in line with the published results on similar material systems^[^ ^98^, ^99^, ^100^, ^101^, ^102^, ^103^ ^]^ and have been explained in complete detail based on microstructural changes at the filler–polymer interfaces,^[^ ^104^, ^105^, ^106^ ^]^ breakdown and reaggregation of the filler clusters,^[^ ^105^, ^106^, ^107^ ^]^ removal of chain entanglements,^[^ ^108^ ^]^ and the slippage/sliding of polymer chains over the filler surfaces.^[^ ^103^, ^105^, ^106^ ^]^ Interestingly, the behavior of the gradient filament is more akin to that of the pure PDMS or PC0. The gradient filament undergoes most of its stress softening at the second cycle with little permanent deformation over multiple strain cycles, similar to the EM‐rich PC20, albeit at a much smaller level.

In addition to evaluating each filament section separately, the entire filament was also subjected to a known uniaxial strain applied at the boundary to observe the strain distribution along its length using two different methods. In the first method, the gradient filament was marked at three equidistant points, virtually dividing it into four segments (see the method in Section 4 and Figure 4c). The heterogeneous strain distribution measured based on the boundary displacement of each segment is shown in Figure 8d. A significant and steady decrease in strain from segments with lower (section #1) to higher (section #4) EM content is clearly visible. Similar to the elastic modulus, the magnitude of the local strain gradient increases drastically upon thermal expansion. While in this method, the gradient filament was assumed to consist of four distinct segments in the digital image correlation (DIC), performed next, we obtained a more finely resolved and continuous strain distribution while the gradient filament was under 10% strain. DIC is a noncontact strain measurement method that compares digital images of objects by tracking speckles or specific patterns/markers on the object's surface before and after deformation.^[^ ^109^, ^110^, ^111^ ^]^ The DIC‐measured longitudinal strain distribution (ε_*xx*_) in the filament, under 10% overall axial strain, is presented in Figure 8e as a heatmap. A gradual increase in local, longitudinal strain from ≈1% on the stiff (high EM‐content) to ≈16% on the soft (low EM‐content) end of the filament is clearly visible. The gradient response corresponds to the gradual decrease in the EM content (Figure 7), making the PDMS matrix increasingly compliant or easier to deform.^[^ ^21^, ^59^, ^96^, ^97^ ^]^ In a similar fashion, the strain distribution in the transverse direction (ε_*yy*_) is the highest on the softer side of the filament, indicating, yet again, the ease of deformation of the side of the filament with lower EM content. In contrast, the strain distribution in the homogeneous filaments PC0 and PC20 under the same overall strain shows a relatively uniform distribution of local strains, see Figure 8f–g. The microscopic variations in strain visible in the homogeneous filaments can be attributed to the inherent variations in materials and processing parameters. Further, readers can refer to multimedia videos S1–S6, Supporting Information, to observe how the local strains vary in the gradient, PC0, and PC20 filaments as the applied global strains gradually increase from 0 to 10%.

**Figure 9:**
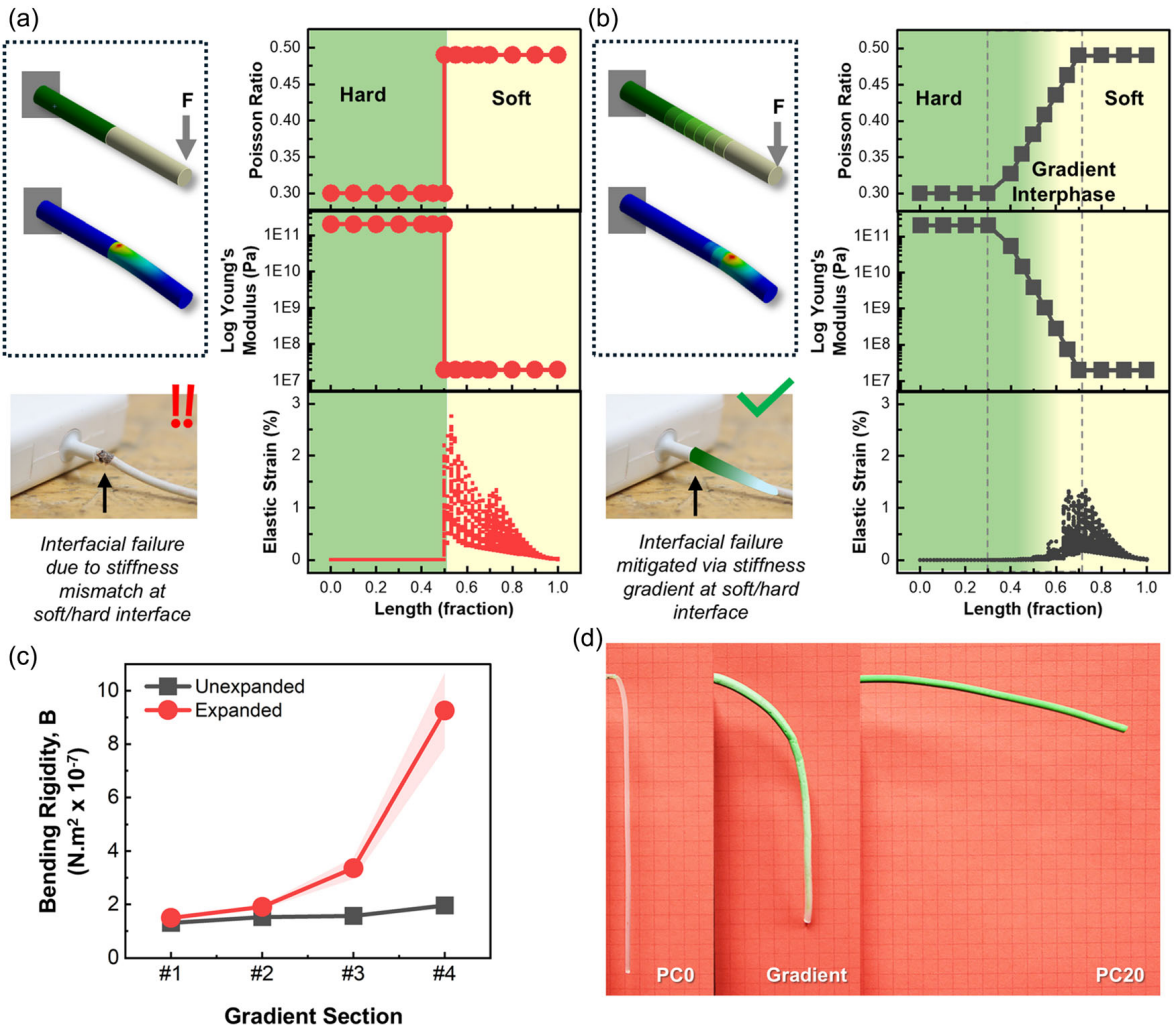
Illustration of elastic strain developed in hard‐soft interface under bending end‐load in the a) absence and b) presence of a gradient inter*phase* between hard and soft phase. A potential application in an electronic charger is shown to elucidate the concept more clearly, c) variation of bending rigidity in each of the four sections of the gradient filament, and d) comparison of bending behavior of the gradient filament under its own weight compared against homogenous filaments PC0 and PC20.

In addition to tensile loads, the interphase of hard and soft materials is likely to experience bending and twisting deformations during use. One of the main causes of interfacial failure in soft materials is repeated flexing (and the resulting fatigue), even when the resulting strain amplitude is much smaller than the rupture strain. The mismatch in response to applied forces or moments may cause the system to fail at the interface due to discontinuity in the response to bending (e.g., curvature, etc.). To illustrate the point, we present a hypothetical situation wherein the soft and hard segments with significantly different material properties (Poisson ratio and Young's modulus) in a cantilever beam are bridged with and without a gradient interphase material. The mechanical response of the cantilever under end‐loading is analyzed using the ANSYS software. In the absence of a gradient interphase, the normal strain abruptly increases at the soft–hard junction (**Figure** **9**a). In contrast, the presence of a gradient interphase mitigates the intensity of strain developed in the body and redistributes the strain away from the interphase toward the compliant/soft side (Figure 9b). An interphase material (e.g., gradient filament) with gradient stiffness in bending in applications, like soft/fiber‐based electronics,^[^ ^29^, ^30^ ^]^ catheters and guidewires,^[^ ^31^ ^]^ tissue scaffolds,^[^ ^33^, ^34^ ^]^ actuators,^[^ ^33^, ^37^ ^]^ etc., is likely to produce smooth and continuous change in curvature and distribute the resulting strain more evenly, such as to mitigate the effect of repeated flexing.

The resistance of a material to bending is expressed as bending (or flexural) rigidity. It describes the material's ability to deform under an applied moment. The flexural rigidity of a soft and flexible beam‐like filament cannot be experimentally characterized easily. Geometric and material nonlinearities are inherent in flexible polymeric materials and structures due to their tendency to undergo large deformations under relatively small loads. While classical beam theories of Euler–Bernoulli^[^ ^112^ ^]^ and Timoshenko^[^ ^113^ ^]^ provide relatively accurate approximations for beams under small deformations, analysis of flexible beams under large deformations requires a more complicated nonlinear approach. In keeping with the limited focus of this research, we demonstrate the gradient nature of the PDMS/EM filament's resistance to bending, assuming linear elastic behavior and using the Euler–Bernoulli beam theory that states that bending moment (*M*) is proportional to the change in curvature (κ), *M* = *B*.κ, and the constant of proportionality (*B* = *E*.*I*) is the bending rigidity, where *I* is the area moment of inertia of the filament cross‐section about the neutral axis, *k* is the curvature of the neutral surface, and *E* is the elastic modulus of the material. The calculated values of *B* from the experimentally obtained dimensions and elastic moduli of the expanded and unexpanded filaments, plotted as a function of filament length in Figure 9c, show the increase in B from the soft to the stiff side of the filament. The significant increase in bending rigidity along the filament length reflects the simultaneous increase in both elastic moduli and diameter of the filament from the soft to the hard side. To further demonstrate the effect of increasing stiffness, the images of three extruded filaments of comparable dimensions but different compositions (homogeneous PC0 and PC20 and gradient) are presented in Figure 9d. The shape of the filaments bent due to their own weight clearly illustrates the effect of the span of the gradient.

While the present research demonstrates the co‐extrusion‐based fabrication for the EM/PDMS material combination, in principle, it applies to other systems that may incorporate gradually changing distribution of fillers, such as metallic/carbonaceous nanoparticle, chemical or physical blowing agents, or hollow glass or ceramic microspheres, within the polymer matrix to obtain desired span and magnitude of the gradient. This adaptability provides a versatile framework for exploring soft mechanical gradients in various material systems.

## 3 Conclusion

Materials and technologies to produce gradient mechanical properties are critical for many current and emerging applications, indeed, it is one of the enabling technologies for the optimum integration of soft and hard materials for structural as well as many functional applications. In this article, we present a co‐extrusion‐based technique that allows controlled distribution of the constituents of a heterogeneous structure within a composite extrudate. We use this technique to produce flexible filaments with a preset gradient design using a thermally responsive EM as fillers within an elastomeric matrix. We shape the stiffness‐gradient EM‐elastomer composite into a high‐aspect ratio filamentous form for applications such as e‐textiles, biomedical devices, and tissue engineering that require gradual stiffness transitions along the length of a wire‐like structure. While the filament morphology shows a core‐sheath structure because of the nature of the static mixer used here, the morphological variation within the filament clearly indicates a gradual increase in the EM content along its length. The corresponding and proportional variation in mechanical properties in unexpanded and expanded filaments was confirmed through experimental determination of Young's modulus and strain distribution along the length of the filament. We determined the gradient nature of the filament through separate tensile tests of segments of filaments, as well as by measuring the strain distribution while the whole filament was held at different strain levels. For a finer resolution of strain distribution, we utilized the DIC method. As intended, the increase in filler content along the filament length increased its initial modulus and correspondingly produced lower strain when the whole filament was held under a constant boundary strain. The magnitude of the gradient due to compositional variation was further enhanced due to the improved filler–filler and filler–polymer interactions upon in situ thermal expansion of the EM particles. Interestingly, the differential dimensional changes after expansion also helped increase the magnitude of the gradient, especially in the bending. The magnitude of the gradient can be manipulated by changing the EM expansion temperature, allowing novel postfabrication tunability. The work reported here presents a simple and practical paradigm enabled by the in‐line mixing and co‐extrusion of commercially available materials to produce soft and lightweight gradient structures having long spans to seamlessly bridge soft‐to‐hard interfaces, thereby benefiting a variety of applications like spanning electronics, medicine, tissue engineering, sensing/actuation, and soft robotics, amongst others.

## 4 Experimental Section

4.1

4.1.1

##### Materials

The EM, Expancel 920 DU 40, having diameters in the range of ≈8–16 μm and operating temperature between 123–168 °C, was generously provided by Nouryon. The thermoplastic shell was a copolymer of acrylonitrile, methyl methacrylate, and methacrylonitrile, while the volatile core was isopentane. The RT curable, platinum‐cured two‐part PDMS elastomer Ecoflex‐0030, with a gelation time of 45 min, curing time of 4 h, and cured tensile modulus (at 100%) of 0.06 MPa, was purchased from Smooth‐On, Inc.^[^ ^114^ ^]^ Part A contained the oligomer and Pt‐catalyst, and Part B contained the cross‐linking agent. A 90 mm long static mixer with 16 mixing elements, a 1.9 cc capacity, and a 1:1 mixing ratio was procured from Amazon for the mixing process. For staining different inputs of EM‐PDMS compositions for identification, a Ni–Co‐based green pigment (Pigment Green 50) was used. All the materials were used as obtained.

##### Methodology

A customized two‐inlet co‐extrusion system was setup to fabricate gradient filaments, see Figure 4a. Two 5 mL standard general‐use syringes containing uncured EM‐PDMS composite with 0 wt% EM (PC0, translucent) and 20 wt% EM (PC20, stained green) were mounted on two separate digital syringe pumps. The two syringe pumps (Genie Touch; GENIE 207359 and GT1035) could support automatic throughput variation capabilities based on the syringe diameter and the plungers’ linear velocities. The two syringes were connected to the inlets of the static mixer (or simply mixer) via two polyethylene (PE) tubes (ID ≈ 1.8 mm). Finally, the extrusion tip of the mixer was connected to a tubular collection mold made of PE (L = 120 mm, ID ≈ 2.0 mm). By precisely controlling the relative throughput ratios of inlet 1 (PC0) and inlet 2 (PC20) of the static mixer using the syringe pump, the proportions of PC0 and PC20 could be programmably and precisely controlled to obtain the desired gradient. Once extruded, the collection mold containing the uncured gradient extrudate was removed and cured in a convective oven (Fisher Scientific Isotemp) for 30 min at 40 °C. Thereafter, the mold was cooled to room temperature, and the filament was demolded. The gradient filaments were ≈100mm long. The unexpanded (as‐extruded) and expanded (130 °C, 30 min) filaments were characterized for their morphological and mechanical behavior.

The morphology of the filaments was examined for the presence of a gradient using optical micrographs of cross and longitudinal sections obtained using a digital Keyence VHX‐E500 microscope. The macroscopic variation in thickness of the expanded filaments could not be measured using a thickness gauge because of their potential distortion even under nominal pressure. Therefore, to ensure reliable measurements, the filament diameter values were measured using the cross‐sectional images via ImageJ software.

The gradient stiffness of the filaments was evaluated experimentally using two methods. In the first method, both the unexpanded and expanded filaments were divided into four longitudinal segments and subjected to tensile tests individually. In the second method, the entire length of the (expanded) filament was subjected to a tensile test. Uniaxial tensile tests were conducted on a tensile testing machine (MTS 30/G) with a 100 N load cell using a cross‐head speed of 250 mm min^−1^. The stress values were calculated using the mean diameter of the gradient segments. The stress–strain curves of the segments were subsequently compared with each other and with the homogenous filaments containing 0 and 20 wt% EM (referred to as PC0 and PC20). Additionally, the strain distribution along the entire filament length was measured by applying three equidistant and distinct marks along its undeformed length and then subjecting it to 100% and 200% global strain, shown in Figure 4c. For a more precise and high‐resolution measurement of strain distribution, and thereby, variation of local stiffness, DIC, an image‐based noncontact strain measurement method, was used on PC0, PC20, and gradient filament samples using MATLAB by implementing the open‐source Ncorr program. High‐resolution images of speckled filament samples were captured at 1, 2, 5, 7, and 10% strain (applied on a load frame (MTS)). Thereafter, the speckle patterns in the strained PC0, PC20, and gradient filament were compared against their respective reference image at 0% strain to calculate strain along the longitudinal (ε_*xx*_) and transverse (ε_*yy*_). To make distinct speckles on the filaments’ surface, a mixture of silicone and carbon black was used in the uncured form. The images of filaments at high strains (>10%) were increasingly difficult to analyze because of the high aspect ratio of the filament and the relatively smaller number of pixels in the cross direction. To simulate the bending of the cantilever with and without a gradient interface between the hard and soft segments, the static structural analysis method of ANSYS software was used, while the end‐load was 20 N. It is important to note that all the tests and analyzes in this study were performed on three specimens from the sample. While we plotted the mean value of the specimen characteristics in all cases, we also plotted an error bar of ±1 SD to represent the variation in the data.

## Conflict of Interest

The authors declare no conflict of interest.

## Author Contributions

**Tushar K. Ghosh**: conceptualization (equal); formal analysis (supporting); funding acquisition (lead); methodology (supporting); project administration (lead); resources (lead); supervision (lead); visualization (supporting); writing—review & editing (lead). **Akanksha Pragya**: conceptualization (equal); data curation (lead); formal analysis (equal); investigation (equal); methodology (equal); software (lead); validation (equal); visualization (lead); writing—original draft (lead); writing—review & editing (supporting).

## Supporting information

Supplementary Material

## Data Availability

The data that support the findings of this study are available from the corresponding author upon reasonable request.
