# Supplementary figures and images for: Extrusion of Heterogeneous Filament‐like Structures: A New Paradigm in Fabricating Soft Mechanical Gradient with Long Span
Source: Small Sci. 2025 May 20;5(8):2500234. doi: 10.1002/smsc.202500234 (PMC12362831; doi:10.1002/smsc.202500234)

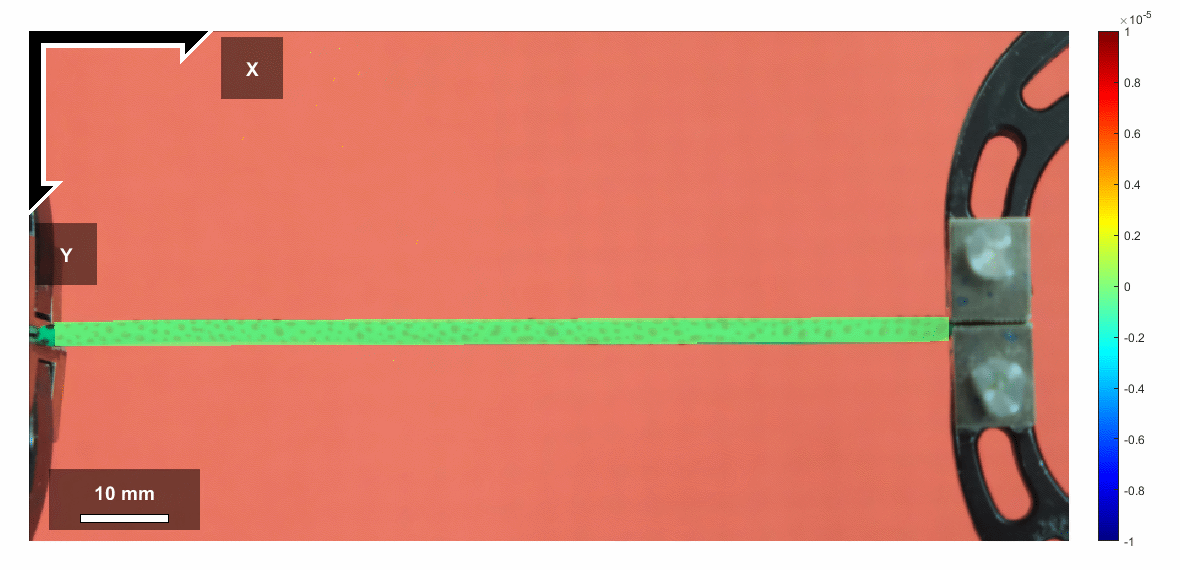

Supplement: Supplementary file 1 — Supplementary Material [file SMSC-5-2500234-s001.zip › smsc.202500234-sup-0001-suppdata-S6.gif]

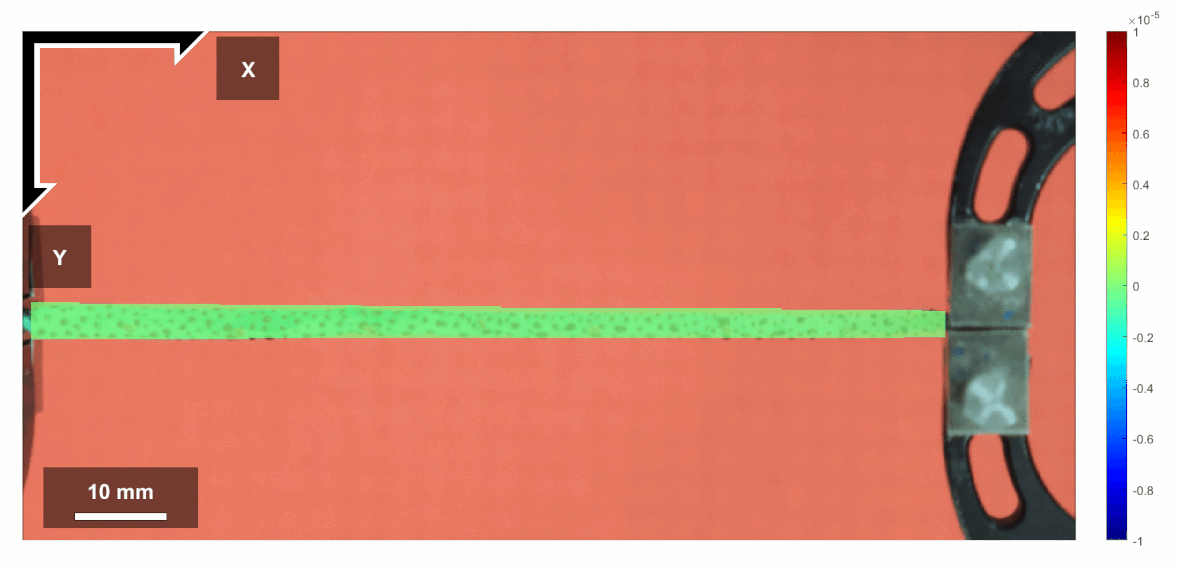

Supplement: Supplementary file 1 — Supplementary Material [file SMSC-5-2500234-s001.zip › smsc.202500234-sup-0001-suppdata-S1.gif]

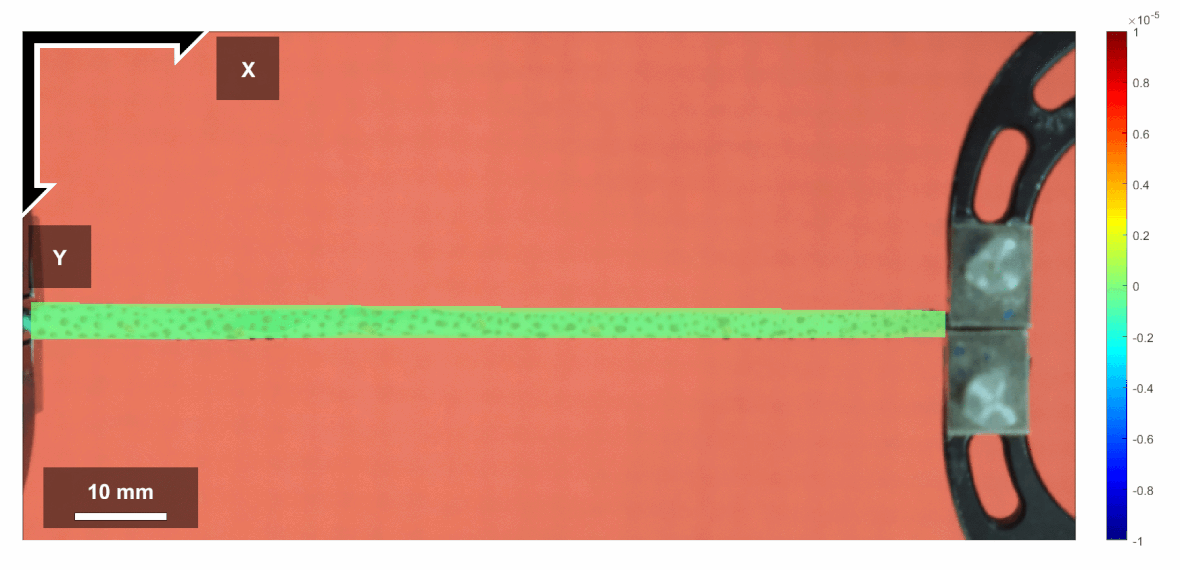

Supplement: Supplementary file 1 — Supplementary Material [file SMSC-5-2500234-s001.zip › smsc.202500234-sup-0001-suppdata-S2.gif]

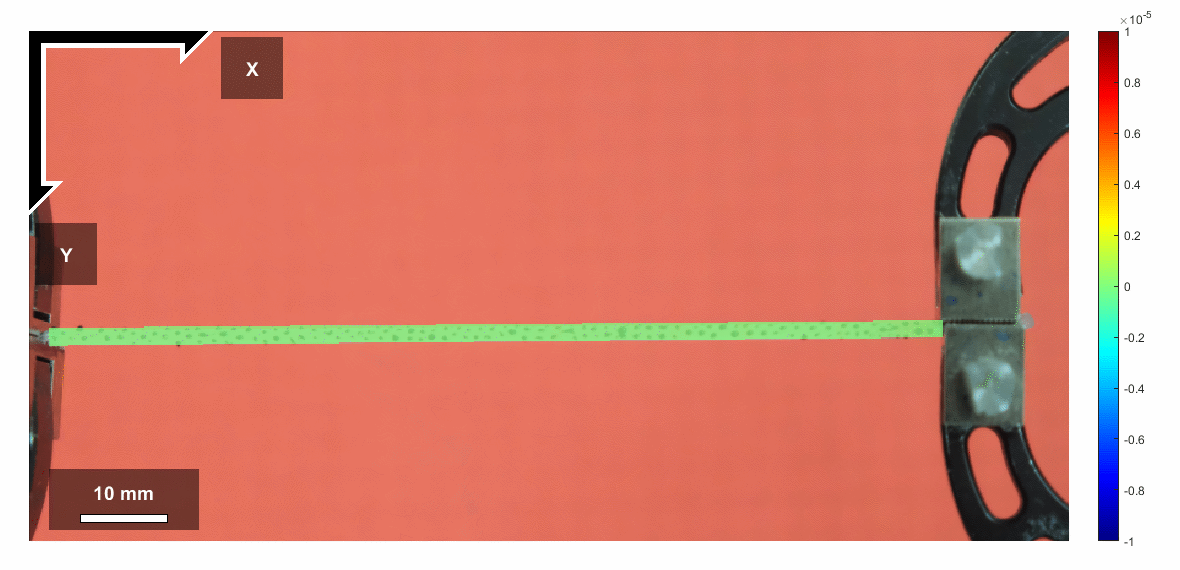

Supplement: Supplementary file 1 — Supplementary Material [file SMSC-5-2500234-s001.zip › smsc.202500234-sup-0001-suppdata-S3.gif]

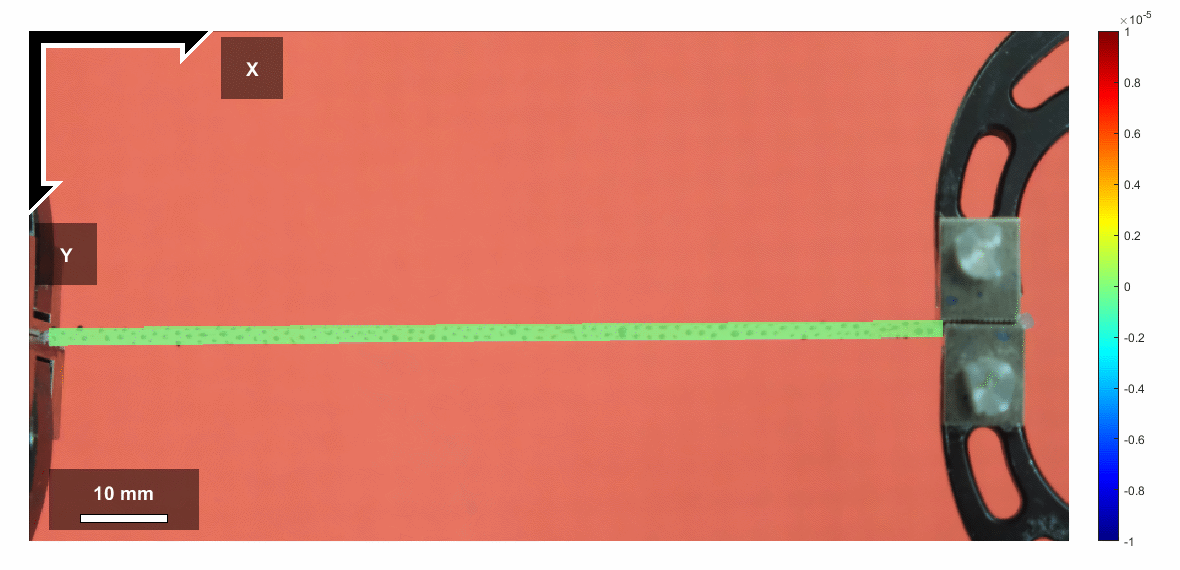

Supplement: Supplementary file 1 — Supplementary Material [file SMSC-5-2500234-s001.zip › smsc.202500234-sup-0001-suppdata-S4.gif]

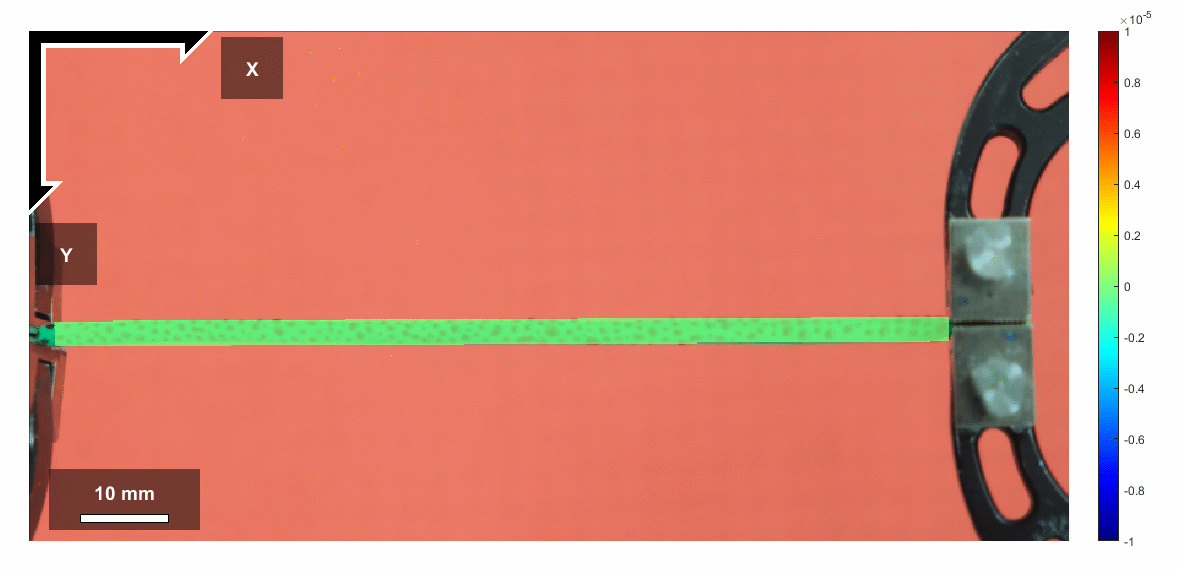

Supplement: Supplementary file 1 — Supplementary Material [file SMSC-5-2500234-s001.zip › smsc.202500234-sup-0001-suppdata-S5.gif]
